# Effects of Predictability of Load Magnitude on the Response of the Flexor Digitorum Superficialis to a Sudden Fingers Extension

**DOI:** 10.1371/journal.pone.0109067

**Published:** 2014-10-01

**Authors:** Ettore Aimola, Maria Stella Valle, Antonino Casabona

**Affiliations:** Department of Bio-Medical Sciences, Section of Physiology, University of Catania, Catania, Italy; Università di Trento, Italy

## Abstract

Muscle reflexes, evoked by opposing a sudden joint displacement, may be modulated by several factors associated with the features of the mechanical perturbation. We investigated the variations of muscle reflex response in relation to the predictability of load magnitude during a reactive grasping task. Subjects were instructed to flex the fingers 2–5 very quickly after a stretching was exerted by a handle pulled by loads of 750 or 1250 g. Two blocks of trials, one for each load (predictable condition), and one block of trials with a randomized distribution of the loads (unpredictable condition) were performed. Kinematic data were collected by an electrogoniometer attached to the middle phalanx of the digit III while the electromyography of the Flexor Digitorum Superficialis muscle was recorded by surface electrodes. For each trial we measured the kinematics of the finger angular rotation, the latency of muscle response and the level of muscle activation recorded below 50 ms (short-latency reflex), between 50 and 100 ms (long-latency reflex) and between 100 and 140 ms (initial portion of voluntary response) from the movement onset. We found that the latency of the muscle response lengthened from predictable (35.5±1.3 ms for 750 g and 35.5±2.5 ms for 1250 g) to unpredictable condition (43.6±1.3 ms for 750 g and 40.9±2.1 ms for 1250 g) and the level of muscle activation increased with load magnitude. The parallel increasing of muscle activation and load magnitude occurred within the window of the long-latency reflex during the predictable condition, and later, at the earliest portion of the voluntary response, in the unpredictable condition. Therefore, these results indicate that when the amount of an upcoming perturbation is known in advance, the muscle response improves, shortening the latency and modulating the muscle activity in relation to the mechanical demand.

## Introduction

The nervous system controls transient, unexpected movements transforming the sensory feedback into a sequence of muscle activity with short-and long-latency reflexes that may be followedby a voluntary response [1], [2]. Many authors have demonstrated that reflexes are more than simple stereotyped responses exhibiting a certain level of adaptation [3]–[7]. The reflex response may be modulated either by endogenous factors such as the muscle tension or the limb position, or by parameters associated with the perturbation such as the dynamics [8], [9] or the direction of the perturbation [4], [10].

Most of these studies have focused on the adaptive relationships between muscle activation and the sensory information delivered with the perturbation. Only few authors have associated the changes in muscle response with information provided before the occurrence of the perturbation. Some investigators have reported shortening of the reflex latency when the mechanical perturbation was anticipated by acoustic [11] or visual [12] signals or by motor imagery [13].

In addition to external signals, motor control may be accomplished by internal information which incorporats the dynamics and/or the timing of the upcoming perturbation. This mechanism typically occurs when the movement is processed by a feedforward controller as in the case of the anticipatory postural adjustments elaborated to prevent the postural instabilities generated by limbs movements [14], [15].

Some studies reported that, if a sudden perturbation occurred during the preparatory period before a voluntary reaching movement, long-latency feedback gain increased in relation to a predictive internal representation of the upcoming reaching dynamics [5], [16], [17]. However, to our knowledge, a direct relationship between predictive information on the mechanical properties of the perturbation and the associated muscle activation has not been investigated yet.

To address this issue, we compared the responses of the Flexor Digitorum Superficialis (FDS) muscle to sudden finger stretching triggered by two different loads during predictable and unpredictable conditions. Our primary expectation was that during predictable condition the timing and/or the gain of the muscle response should improve.

Data presented here would contribute to strengthen the idea that feedback control may be, to some degree, influenced by anticipatory commands and that internal signals integrate peripheral sensory information to optimize reflex response.

## Materials and Methods

### Ethics Statement

A written informed consent was obtained from each participant according to the Declaration of Helsinki and the experimental procedures were approved by the ethics committee of the University of Catania.

### Subjects

Eleven right-handed adults without signs of neurological disorders took part in this study. Two subject were eliminated from all analyses due to the numerous abnormal responses (see below), bringing the total to 9 subjects (4 males and 5 females; 33±7 years; range: 26–46). All were naive to the experimental objectives and the test apparatus.

### Apparatus and procedures

**Figure 1:**
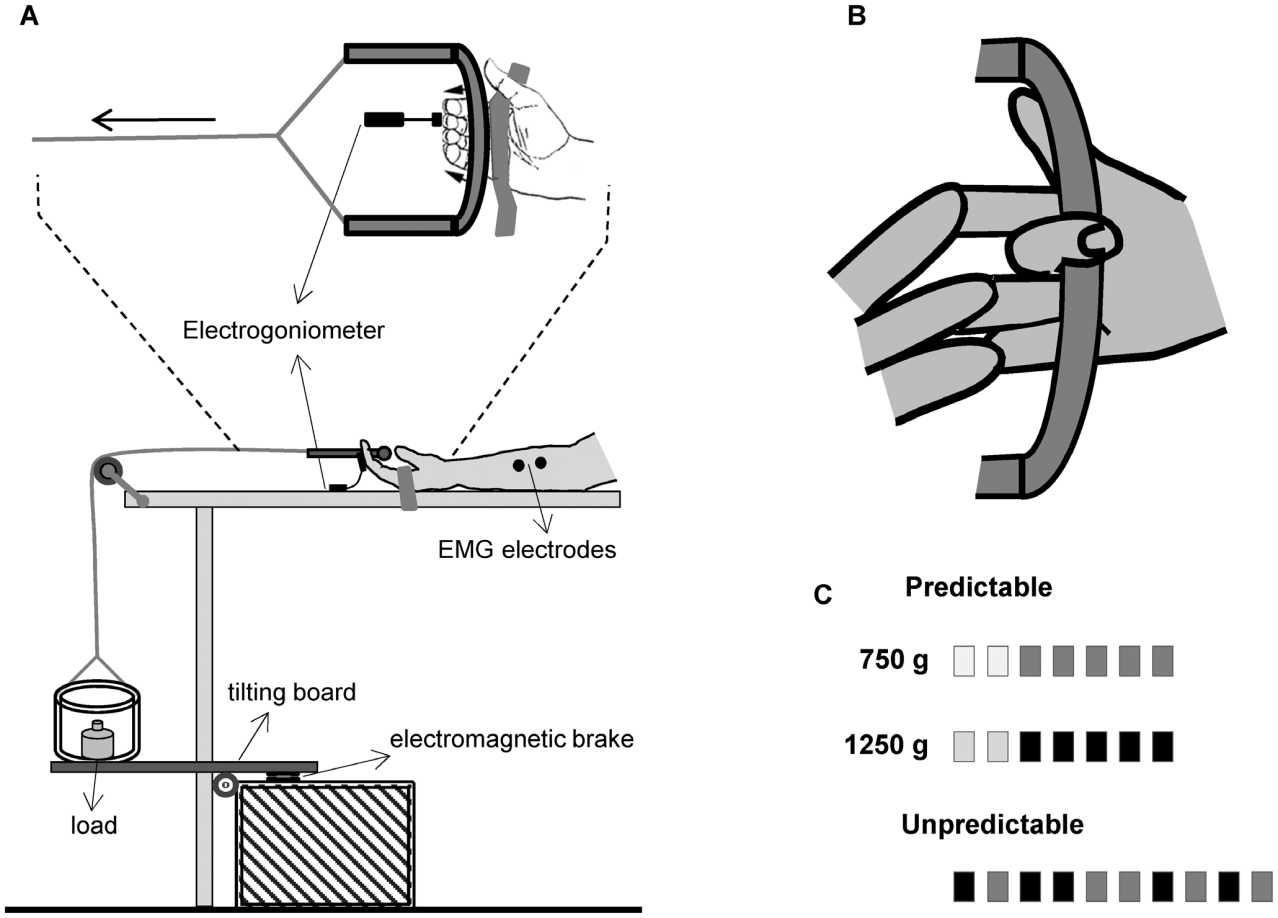
Experimental set-up. (A) Schematic drawing of the apparatus arrangement and experimental task. (B) Procedure to test the FDS muscle. (C) Experimental protocol. The lighter shade for the first two boxes of each predictable sequence indicates the trials not considered for the analysis. For further explanation see the text.

The tests were performed with subjects sat on a chair in a comfortable position with their right forearm and hand resting supine on a table (Fig. 1A). The hand was oriented in the parasagittal plane passing through the shoulder and the table provided a base of support for the fingers rotation. The palm was secured to the table by a belt to restrain metacarpophalangeal and finger joints movements. All the fingers were freely relaxed with the fingers 2–5 placed next to a handle. A 1.5 m, stiff nylon cable connected the handle to a load via a pulley fixed on the table. Two levels of load, either 750 or 1250 g, were used to pull the handle. A tilting board locked to a firm support by an electromagnetic brake, sustained a box that contained the load. To guarantee a smooth handle motion over the parasagittal plane, very low frictional resistance materials were used and the components were carefully secured and aligned along anterior-posterior axis.

The handle was aligned with the base of the middle phalanx of the digit III and positioned 0.5 cm from the palmar surface of the finger. In this way, the stretching of the FDS was maximized as the distal end of the muscle is inserted at the base of the middle phalanx. No background activity was imposed to the muscle by preloading the fingers and, before applying the perturbation, participants were asked to close their eyes and fully relax limb muscles. The finger joint perturbation occurred when an experimenter unlocked the electromagnetic plate allowing the tilting board to rotate and the load to be released. As the load fell, the cable pulled the handle and all four fingers were rapidly extended as a whole. Subjects were instructed to grasp the handle very quickly and to pull it back to around the start position. No warning signal was provided and, for both the conditions, the experimenter released the load after a time interval which varied from 2 to 6 s after subjects closed their eyes. By this setting the differences of predictability between the two conditions were restricted to the load weight. It was checked that the muscle was at rest prior to the perturbation as evidenced by the absence of electromyographic (EMG) activity, subjects did not anticipate the perturbation and did not open their eyes during the test.

The experimental protocol is illustrated in the figure 1B. Subjects performed one block of seven consecutive trials for each load (predictable condition) and one block of ten trials with a randomized distribution of the loads (unpredictable condition). Before starting each block, subjects were informed about the predictability condition of the upcoming trials and, in the case of the predictable condition, they knew in advance the load weight. To reduce the variability due to the initial uncertainty, we eliminated the first two trials of each block performed in the predictable condition and analyzed the following five trials. We balanced the order of presentation of load magnitude and predictability conditions across the subjects. Each trial was monitored on-line to detect abnormal movements or irregular EMG signals: trials showing visible variations of baseline electrical activity or finger movements before occurring the perturbation or large excursions over the frontal plane, were excluded. When the irregularities occurred for no more than two trials across the blocks, new trials were performed. To prevent the effect of practice, subjects which performed incorrect trials more than two times, were excluded from the analysis. This latter was the case of two subjects. One subject was removed because many responses started before the handle movement and the baseline activity exhibited large amplitude variations. In the other case, the subject showed difficulties to maintain a stable full forearm supination. As a consequence he exhibited a high level of baseline activity and, during many trials, a strong traction was exerted on the strap producing lateral finger displacements.

### Kinematics processing

A flexible biaxial electrogoniometer (Biometrics Ltd, Gwent, UK) provided a measure of the finger motion. The distal end of the electrogoniometer was attached to the dorsal face of the middle phalanx of the digit III, while the proximal end was attached to the table (see Fig. 1A). The electrogoniometer placement was arranged so that the anterior-posterior axis was parallel to the sagittal plane. This positioning meant that the anterior-posterior axis measured the change in angle of the proximal interphalangeal joint of the digit III, while the medial-lateral axis captured the finger motion over the frontal plane. We used this latter measurement to detect abnormal frontal excursions of the finger. Although the anterior-posterior axis of the electrogoniometer measured the angular variations of the proximal interphalangeal joint of the digit III, the high synchronization of the finger movements required by our task, allow us considering this measurement as a reliable assessment of the motion of all the fingers.

**Figure 2:**
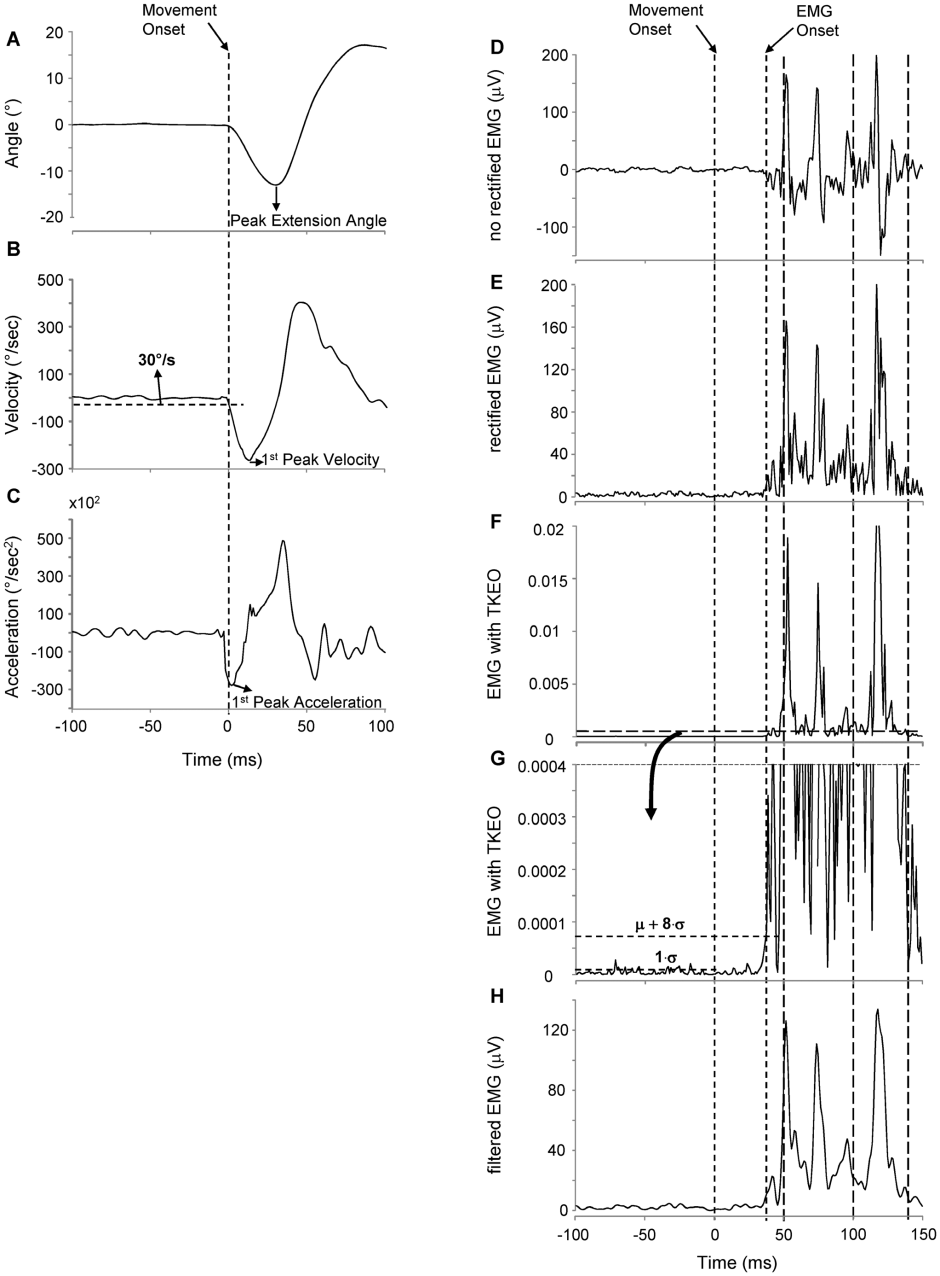
Kinematics of the finger movement and electromyographic conditioning. Kinematic traces of position (A), velocity (B) and acceleration (C) of the finger angular rotation. EMG activity is presented as high pass filtered (D), full wave rectified (E), conditioned with Teager–Kaiser energy operator (F and G) and low pass filtered (H). Vertical dashed lines with large dash length delimit the intervals including short-latency reflex, long-latency reflex and the earliest portion of the voluntary response. μ and σ represent the mean and the standard deviation of the baseline activity over 100 ms prior the movement onset. For further explanation see the text.

The joint angle data were sampled at 1kHz as we collected the EMG and positional data by the same device (PocketEMG by Bioengineering Technology and System, BTS, Milan, Italy). The positional signals were run through a low-pass filter (lag-zero, 2 pass, 4^th^ order Butterworth) with a cut-off determined by performing the residual analysis of the differences between filtered and raw data over a wide range of cut-off frequency. We plotted residual vs low-pass filter cut-off frequency and used this graph to choose the cut-off frequency that separated the noise from the true signal as described by Winter [18]. Data obtained for each experimental section produced cut-off frequencies between 50 and 60 Hz. Thus, the mean value (55 Hz) was chosen to filter all the traces that were further visually inspected to ensure correct attenuation of the raw signal. The filtered angular position trace (Fig. 2A) was numerically differentiated to calculate angular velocity and angular acceleration (Fig. 2B–C).

Movement onset was determined with the use of a criterion of 30°/s increasing for at least 20 ms for the rotation of the digit III (Fig. 2B). For statistical comparisons we determined the peak extension angle (Fig. 2A), the first peak velocity (Fig. 2B), the first peak acceleration (Fig. 2C) after the movement onset and the peak overshoot at the end of the finger flexion.

### Electromyographic processing

Surface EMG was recorded with 11-mm bipolar, Ag-AgCl electrodes (Gereonics Inc., Irvine, CA USA), with an interelectrode distance of 15 mm. The electrodes were placed at midforearm, halfway from the ventral midline to the medial border of the forearm. At this location, the position of the electrodes was carefully adjusted with the aim to maximize the signal from the FDS muscle. The main action of the FDS is to flex the second phalanges over the first around proximal interphalangeal joints of the fingers 2–5. The FDS was tested by asking the subject to flex the digit III at the proximal interphalangeal joint against external resistance while observing and palpating the forearm over the contracting muscle (Fig. 1C). The remaining three fingers are held fully extended reducing the possibility that the flexor digitorum profundus, which inserts into the distal phalanges, was a major contributor to the EMG signal. To minimize the contribution of wrist flexors to the EMG signal, electrodes placement was considered appropriate when the EMG signal was present during isometric finger flexion with a voluntary stable wrist, yet reduced significantly during isometric wrist flexion with no voluntary finger flexion. Since the isometric force was applied by the digit III, the main contribute to the EMG signal should be provided by the portion of FDS serving this finger. Subjects quickly familiarized with the maneuvers, executing correctly the instructions.

The EMG signal was pre-amplified at the electrodes site and sampled at 1 kHz (PocketEMG by Bioengineering Technology and System, BTS, Milan, Italy). The raw EMG signal was first off-line high-pass filtered (20 Hz, 2 pass, 4^th^ order Butterworth) to remove movement artifacts (Fig. 2D), and then it was full wave rectified for further signal conditioning (Fig. 2E).

The onset of the EMG burst was determined using the nonlinear Teager–Kaiser energy operator (TKEO). Compared to other methods that consider only the signal amplitude in determining the onset time, TKEO takes into consideration both amplitude and frequency and computes the energy of the EMG signal. It has been demonstrated that this method improves the signal-to-noise ratio and increases the accuracy of the EMG onset detection [19]–[22].

We determined the onset of muscle activation on a trial-by-trial basis. For each EMG trace, the TKEO was applied on the 20 Hz high pass filtered raw signal (Fig. 2D) and the TKEO output was full wave rectified (Fig. 2F).

The threshold T to identify the onset time over the TKEO domain was determined as follows (see the magnification of the EMG with TKEO in Fig. 2G):
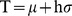
where *µ* and *σ* are the mean and standard deviation of a reference baseline chosen from 100 ms before the finger movement onset; *h* is a preset variable, defining the level of the threshold. We assigned to *h* the value of 8 according to the data from Li et al. [19] and after preliminary tests performed on a sample of our EMG traces. Li et al. [19] found that threshold values ranging between 6 and 8 introduced the minimal detection latency. To adapt the level of *h* to our data, we performed a validation process to test the outputs of thresholds with four values of *h* (4, 6, 8 and 10). A MATLAB routine was written to implement a computerized method which analyzed the EMG signals from a sample of 27 trials selected one for each session, across all the subjects. In parallel, an independent observer determined visually the EMG onset on the rectified no-TKEO sample traces. The onset determination by visually-detection was compared with the TKEO method and the result indicated that a threshold level with *h = *8 introduced the smallest detection error.

On these bases, latency measurement was performed from finger movement onset to EMG response onset. All results were verified by further inspection to detect visible incongruence between the two onsets.

To evaluate the amount of activity associated with the muscle response, the rectified EMG without TKEO (Fig. 2E) was run through a low-pass filter (150 Hz, 2 pass, 4^th^ order Butterworth; Fig. 2H) with the cut-off determined as described in the previous section “*Kinematics processing”.*

Mean EMG activity was computed on the filtered EMG over three temporal windows from the onset of the finger extension (see vertical green lines in Fig. 2D–H): the first interval, from EMG onset to 50 ms from the movement onset, included the earliest EMG activity (short-latency reflex); the second temporal window, including EMG activity from 50 to 100 ms from movement onset, represented non voluntary long-latency response (long-latency reflex); the third interval, from 100 to 140 ms from the movement onset, displayed the initial portion of the voluntary response. The criterion for this intervals partition was based on evidences from many authors which reported, for the hand muscles, latencies below about 50 ms for the short-latency reflex and above about 100 ms for the initiation of the volitional reaction [1], [2], [13], [23]–[25]. These time epochs were appropriately modified when a more detailed description of the muscle activity was required.

Mean muscle activity within each temporal window was determined by performing the following step: first, the mean level of the baseline activity was calculated over 100 ms before the onset of the perturbation; second, the mean baseline level was subtracted from the mean EMG of each temporal interval to obtain the net EMG activity; third, the net EMG activity was normalized by the mean baseline level and the EMG amplitude was expressed as baseline unit (bu). Normalized mean values were computed trial-by-trial and single subject average was calculated. A grand average across subjects was obtained and used to compare the EMG activity between loads and predictability conditions.

All filtering in our analyses was run forward and reverse to eliminate phase lag.

### Statistical analysis

Statistical comparisons were performed by a repeated measures ANOVA using two levels of load (750 vs 1250 g) and two levels of predictability (predictable vs unpredictable) as within-subject factors. Repeated measures ANOVA was also used to compare amounts of EMG activity across six timing intervals (two levels of load and six levels of interval) and to compare the latency across unpredictable (two levels of load and seven levels of trial) and predictable (ten levels of trial) temporal sequence of single trials. In these cases the critical value of F was adjusted applying Greenhouse-Geisser correction which produces a p-value more conservative. This procedure corrects the repeated-measures ANOVA with respect to a possible violation of the sphericity assumption, that is, the variance of the differences among all combinations of independent variables must be equal [26]. ANOVAs were followed by Bonferroni-corrected pairwise comparisons to evaluate differences in performance between single levels. Paired Student’s t-test was used when appropriate.

The level of significance was set to p<0.05 and the results are presented as mean ± standard error.

Signal analysis was performed by Matlab, version R2012a (Mathworks Inc, Natick, MA, USA) and statistical analysis was performed by SYSTAT, version 11 (Systat Inc., Evanston, IL, USA).

## Results

**Figure 3:**
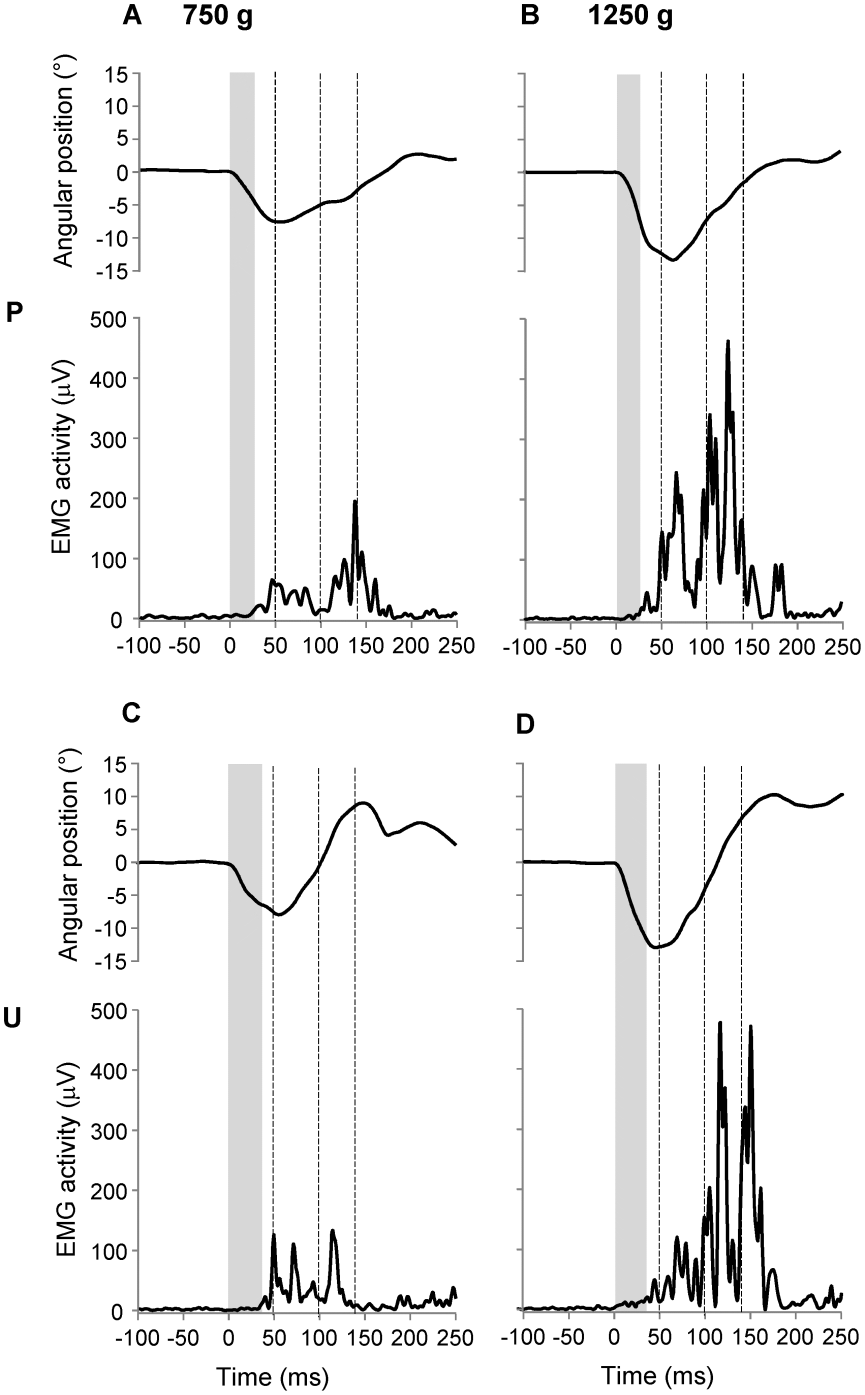
Examples of finger displacements and EMG responses. Angular position and EMG traces from single trials of one representative subject performing predictable (A–B) and unpredictable (C–D) conditions with load of 750 g (A–C) or 1250 g (B–D). Traces have been aligned with the onset of the finger movement (time 0) and the vertical shadow areas demarcate the time interval between the onset of finger movement and the onset of EMG activity. Vertical dashed lines with large dash delimit the same intervals as in Fig, 2. U, unpredictable condition; P, predictable condition.

Muscle activation and finger angular displacement are shown in the Fig. 3 for a representative subject. Panels show trials from predictable (P; Fig. 3A–B) and unpredictable (U; Fig. 3C–D) conditions performed using pulling loads of 750 g (Fig. 3A and Fig. 3C) and 1250 g (Fig. 3B and Fig. 3D). These examples capture three typical behaviors observed across the subjects: the angular excursion during the finger stretching (downward direction in the angular position plots) increased from the lighter to the heavier load; the latency of EMG response was shorter in predictable than unpredictable condition (see width of the grey areas); the level of muscle activation showed a parallel increase with the load weight, starting from about 50 ms for the predictable trials and from about 100 ms for the unpredictable trials.

These features also emerged from the statistical analysis of temporal and amplitude parameters performed across the subjects with respect to the two loads and predictability conditions.

### Changes in the finger kinematics

**Figure 4:**
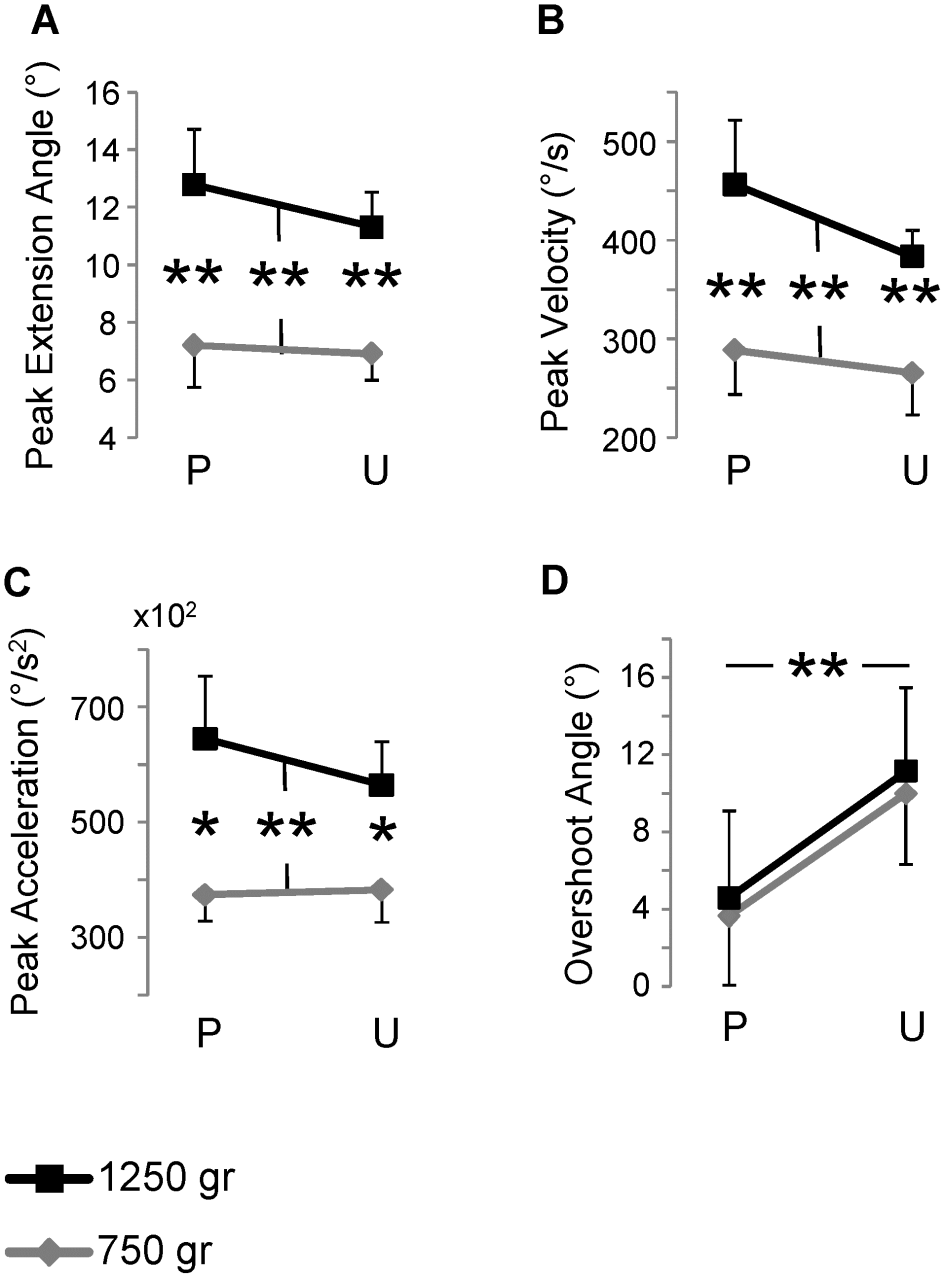
Kinematics analysis. Means and standard errors of the peak extension angle (A), first peak velocity (B), first peak acceleration (C) and the overshoot angle (D). The data in the graphs result from the grand average across subjects over the five trials performed for each load during predictable (P) and unpredictable (U) condition. ** *p*<0.01; * *p*<0.05. The symbol * or ** embraced between two lines indicates main effect detected by ANOVA while, in the other cases, the level of significance indicates simple effect assessed by paired t-test.

Load variations influenced greatly all the kinematic parameters measured during the finger stretching after the perturbation (Fig. 4). A significant increase with the load was exhibited by the peak extension angle (*F*_1,8_ = 47.03; *p*<0.001; Fig. 4A) both in predictable (7.2±1.5° for 750 g and 12.8±1.9° for 1250 g; *t*_8_ = 7.49; *p*<0.001) and unpredictable condition (6.9±0.9° for 750 g and 11.3±1.2° for 1250 g; *t*_8_ = 3.64; *p* = 0.007), by the peak velocity (*F*_1,8_ = 33.23; *p*<0.001; Fig. 4B) both in predictable (288.2±44.5°/s for 750 g and 455.2±65.2°/s for 1250 g; *t*_8_ = 3.64; *p* = 0.007) and unpredictable condition (265.4±42.2°/s for 750 g and 382.8±26.7°/s for 1250 g; *t*_8_ = 3.47; *p* = 0.008) and by the peak acceleration (*F*_1,8_ = 20.11; *p* = 0.002; Fig. 4C) both in predictable (37560±4525°/s^2^ for 750 g and 64565±10872°/s^2^ for 1250 g; *t*_8_ = 3.01; *p* = 0.017) and unpredictable condition (38429±5592°/s^2^ for 750 g and 56530±7499°/s^2^ for 1250 g; *t*_8_ = 3.29; *p = *0.011 ), whereas there was no load predictability main effect or significant interaction between load and predictability conditions for all the parameters. Conversely, the magnitude of the overshoot movement occurring at the end of the finger flexion changed significantly between the two predictability conditions (*F*_1,8_ = 34.38; *p*<0.001; Fig. 4D) with the lighter load increasing from 3.7±3.6° to 9.9±3.7° (*t*_8_ = 3.62; *p = *0.007) and the heavier load increasing from 4.6±4.5° to 11.1±4.3° (*t*_8_ = 3.04; *p = *0.016). No significant difference was observed between the loads or for the load-predictability interaction.

### Analysis of the latency of EMG responses

**Figure 5:**
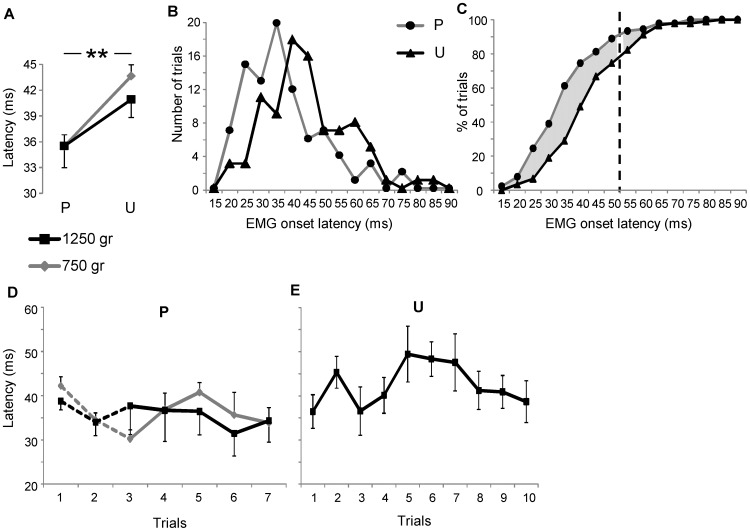
EMG onset latency analysis. (A) Means and standard errors of the latency of EMG responses. Distribution (B) and cumulative (C) frequency histograms of the latency of EMG responses measured during predictable and unpredictable conditions regardless of the load. The grey area between the cumulative histograms indicates the fraction of responses with delayed latency during unpredictable compared with predictable condition before and after 50 ms from the movement onset (vertical dashed line). Latency values across the temporal sequences of predictable (D) and unpredictable (E) trials. Symbols and abbreviations are the same as in Fig. 4.

The latency of EMG responses exhibited significant differences in relation to the predictability of the load (*F*_1,8_ = 19.78; *p* = 0.002; Fig. 5A), increasing from predictable (35.5±1.3 ms for 750 g and 35.5±2.5 ms for 1250 g) to unpredictable condition (43.6±1.3 ms for 750 g and 40.9±2.1 ms for 1250 g), but the main effect of the load and the interaction between the two factors were not significant. A further quantification of the latency of EMG response was provided by analyzing the latency distribution over predictable and unpredictable trials, regardless of the load (Fig. 5B–C). The latency distribution during unpredictable trials shaped a less skewed histogram than that produced by the values of latency measured in the predictable condition (35.0±4.7 ms and 42.5±4.8 ms for predictable and unpredictable condition, respectively; Fig. 5B). The cumulative plot (Fig. 5C) shows that most of the lagged EMG responses during unpredictable condition occurred below 50 ms from the movement onset (about 70%; grey area to the left of the vertical dashed line in Fig. 5C), while delayed responses with latency above 50 ms were only about 15% (grey area to the right of the vertical dashed line in Fig. 5C). Latency values were also plotted in relation to the temporal sequence of trials during predictable (Fig. 5D) and unpredictable condition (Fig. 5E). All the sequences exhibited no significant trends across the trials, except for the local comparison between trials 1 and 2 for the lighter load, during predictable condition (*p* = 0.024 with Bonferroni correction).

### Analysis of the changes in the level of EMG activity

**Figure 6:**
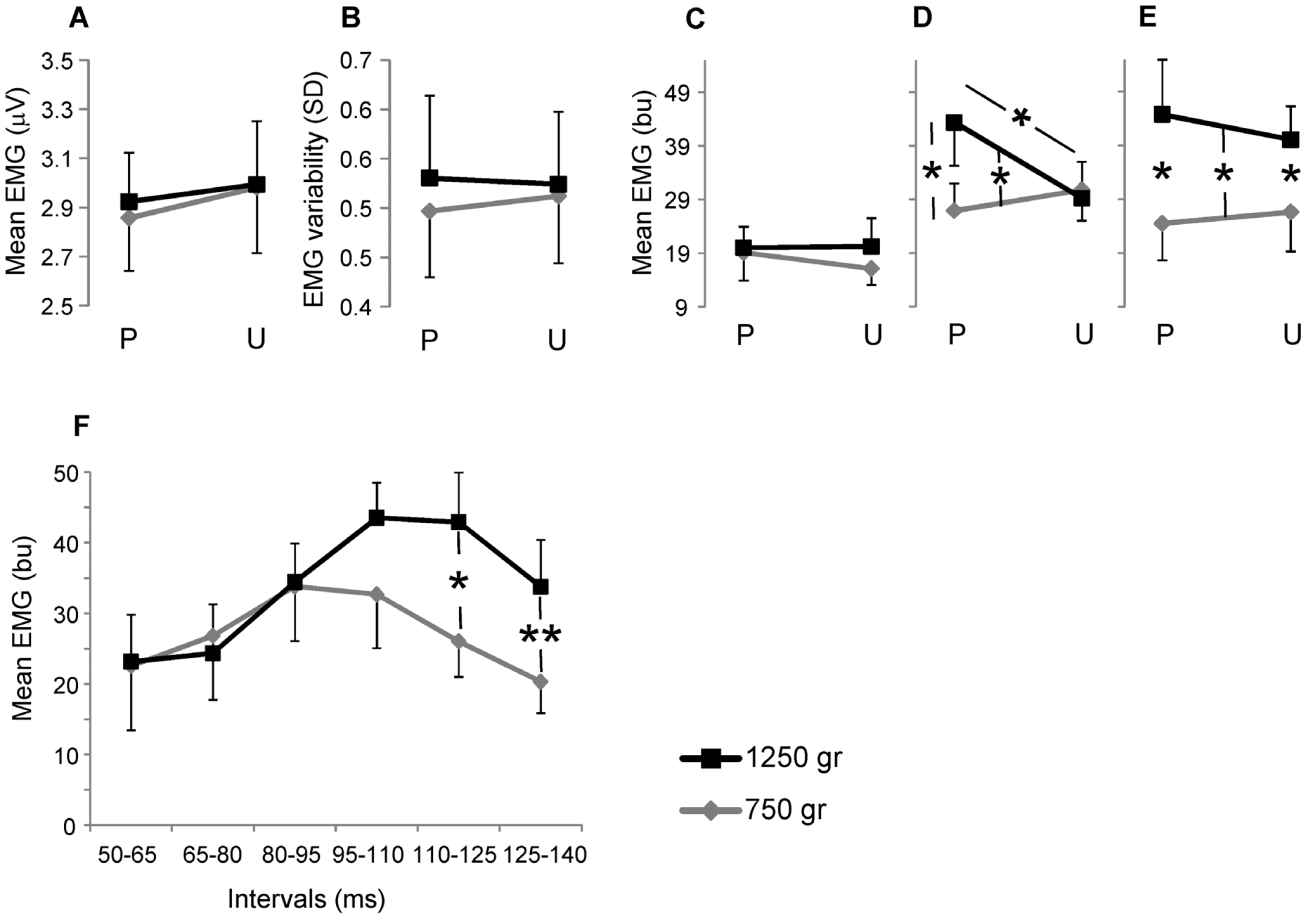
Electromyographic activity analysis. Mean level of baseline activity (A) and its variability (B) recorded over 100 ms prior the movement onset. Normalized mean level of EMG activity during short-latency reflex (C), long-latency reflex (D), initial portion of the voluntary response (E), and unpredictable condition computed with intervals of 15 ms between 50 to 140 ms from the movement onset (F). Symbols and abbreviations are the same as in Fig. 4.

The Figure 6 illustrates the variations of the level of EMG activity with respect to the load and the predictability conditions. The mean baseline level (Fig. 6A) and its variability (Fig. 6B) were unchanged in relation to all the factors. There was no significant variation also for the mean EMG activity between the onset of the muscle activation and 50 ms after the movement onset (short-latency reflex; Fig. 6C). A significant interaction was observed between load and predictability for the mean EMG activity recorded between 50 to 100 ms from the movement onset (long-latency reflex; F_1,8_ = 8.94; *p* = 0.017; Fig. 6D): the gap between the two levels of normalized EMG activity relative to load magnitudes, clearly increased from unpredictable (30.8±5.2 bu for 750 g and 29.2±4.1 bu for 1250 g) to predictable condition (26.9±5.1 bu for 750 g and 43.3±8.0 bu for 1250 g). The effect of the load was also significant (F_1,8_ = 5.49; *p* = 0.047), but the simple comparison between the loads was significant only for the predictable condition (*t*_8_ = 3.15; *p* = 0.014). No statistical changes were observed for the predictability factor. Over the time between 100 and 140 ms after movement onset (initial portion of the voluntary response; Fig. 6E) the EMG activity showed significant differences only in relation to the loads (F_1,8_ = 7.19; *p* = 0.028), both for predictable (24.5±6.8 bu for 750 g and 44.8±10.2 bu for 1250 g; *t*_8_ = 2.32; *p* = 0.048) and unpredictable condition (26.6±7.3 bu for 750 g and 40.1±6.2 bu for 1250 g; *t*_8_ = 2.54; *p* = 0.035). A more detailed temporal analysis over the interval between 50 to 140 ms from the movement onset, was performed reducing the single window time interval from 40 to 15 ms (Fig. 6F). In this case there was only the main effect of the interaction between loads and intervals (F_1,40_ = 3.54; *p* = 0.035 with Greenhouse-Geisser correction). The unpredictable condition showed a progressive increase of the differences in EMG activity between the two loads, with significant changes in the temporal windows ranging from 110 to 125 ms (*t*_8_ = 3.2; *p* = 0.013) and 125 to 140 ms (*t*_8_ = 3.52; *p = *0.008).

**Figure 7:**
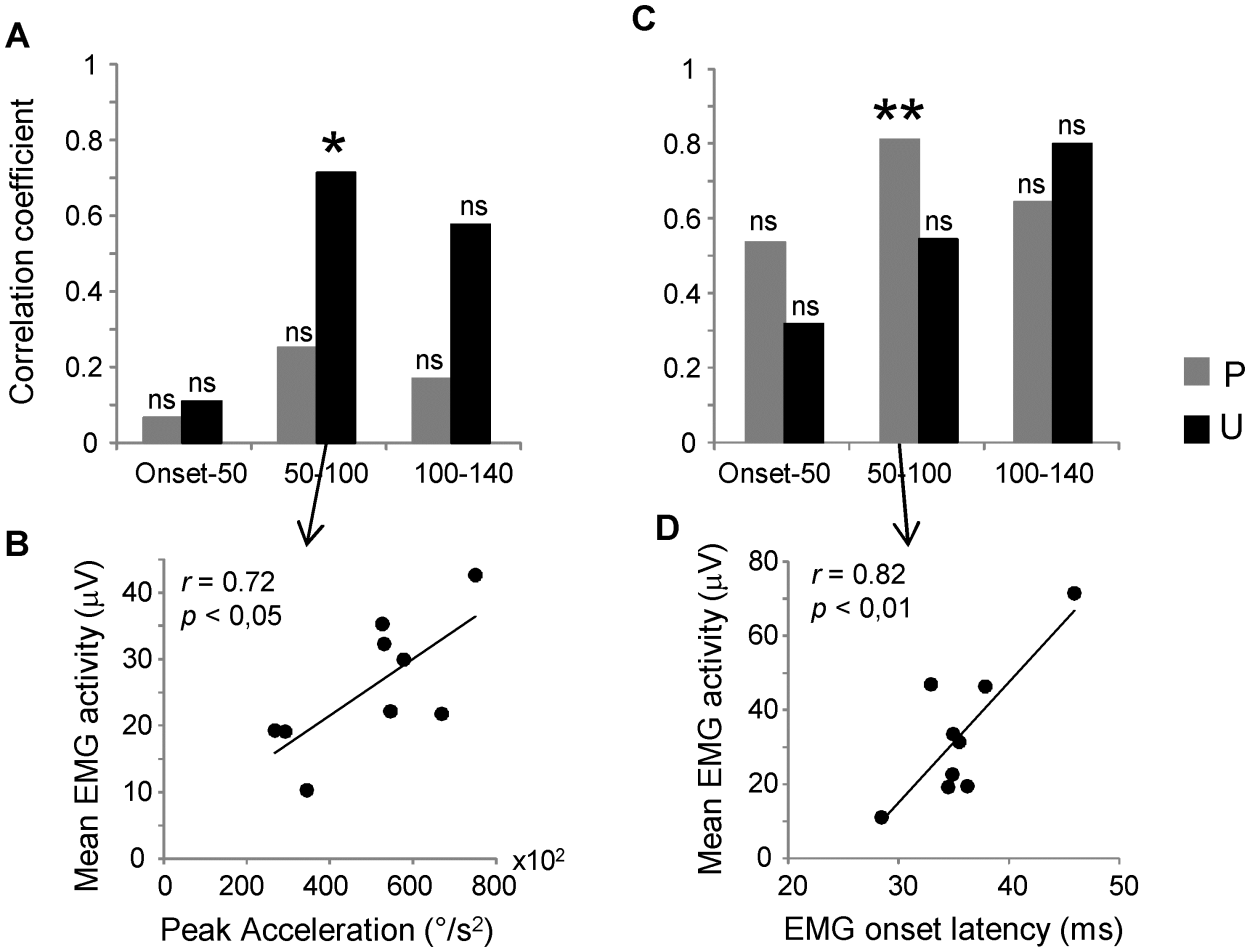
Correlations of mean EMG activity with kinematic and EMG onset latency. Relationships between mean EMG activity and peak acceleration (A–B) and between mean EMG activity and EMG onset latency (C–D) over the three temporal windows. The regression plots (B–D) are reported only for the correlations displaying a level of significance with *p*<0.05. Each data point results from the average computed in each subject over ten trials performed during predictable (P) and unpredictable (U) conditions regardless of the load level. ** *p*<0.01; * *p*<0.05; ns: not significant.

To ascertain possible relationships between EMG activity and kinematic or temporal data over the three predefined time windows, we performed linear regressions comparing the mean level of EMG activity with the peak acceleration (Fig. 7A–B) and with the EMG response latency (Fig. 7C–D). The analysis of variance showed significant correlations only in the interval between 50 and 100 ms, with good correlation coefficients for the relationships between EMG level and peak acceleration in the unpredictable condition (*r* = 0.72; *p* = 0.031; Fig. 7A–B) and between the EMG level and latency in the predictable condition (*r* = 0.82; *p* = 0.009; Fig. 7C–D). Several of the other relationships exhibited acceptable correlation coefficients but the level of the statistical assessment was not significant.

## Discussion

In the present set of experiments, the FDS muscle activity evoked by sudden finger stretching showed temporal and amplitude changes in relation to the predictability of the load: when the subjects did not know in advance the weight magnitude, the latency of the EMG response lengthened and the level of muscle activation matched the load weight late after the perturbation.

### Temporal changes in muscle response

Many studies report that, when subjects are instructed to actively react to a rapid joint displacement, a sequence of responses occurs in the muscles that are stretched [3], [23], [24], [27], [28]. In the case of finger muscles, the earliest muscle activation, compatible with the spinal stretch reflex, is observed below 50 ms from the mechanical perturbation. Latency of cutaneous reflexes may also have short timing [25]. A late component, between 50 to 100 ms, might involve transcortical loop [24], [29]–[31], but it is fast enough to be considered a reflex response. Some authors suggested that a fraction of this component may be ascribed to spinal circuits associated with afferences provided by group II muscle spindle afferents [32] or cutaneous mechanoreceptors [33]. The voluntary muscle activation can occur 100 ms after the movement onset [3], [27], [28].

Since most of the delayed muscle responses during unpredictable condition started below 50 ms from the movement onset (about 70%), the temporal changes observed here should involve all the components of the EMG response including the short latency reflex. For the fraction of lagged responses with latency above 50 ms (about 30%), the circuits which control short latency reflexes should be excluded. Small fractions of responses (25% in predictable and 5% in unpredictable condition) showed fast response with latency below 25 ms. This value, reported also by other authors [13], [27], [34], may depend on differences on subjects height as the spinal reflex are correlated to arm length [35]. In fact, most of responses below 25 ms were recorded in two subjects with the lowest height.

Shortening in response latency within the timing interval of the short-latency reflex was observed when a quick joint perturbation was anticipated by motor imagery [13], acoustic [11] or visual [12] cues. To our knowledge this is the first study in which the latency of muscle response, including the short-latency component, was associated with the load predictability.

Although latency variations were not influenced by the load magnitude, they were strongly correlated with the level of mean EMG activity recorded in predictable condition during the temporal window of the long-latency reflex (fig. 7C–D). This relationship would imply that, at least for the shortest EMG responses, predictive information on the perturbation dynamics influenced the spinal circuits serving short latency reflex, but the mechanical consequences of this early effect were displayed later during the timing of the long-latency reflex.

A possible explanation for the changes in latency may be that a predictive load-dependent signal modulated the subthreshold excitation of motoneurons, reducing or increasing the activation time when the sensory afference impinges upon the motoneurons (see [36]). Therefore, in the predictable trials a feedforward modulation of the motoneurons excitability reduced the time for the sensory input to reach the threshold of activation. On the contrary, during the unpredictable condition the state of the membrane excitation was left at a baseline level, forcing the sensory signal to follow a simple feedback circuit that delayed the muscle activation. Although our experiments did not provide direct evidences to support this interpretation, a consolidate literature reporting extensive neuronal modulation at several locations in the spinal cord (for a recent review see [37]) and some studies suggesting that the spinal cord may incorporate pre-movement instructions [38] and internal models of limb dynamics [39], make it likely that the timing changes in the short-latency reflex depended on a predictive signal linked to the level of load magnitude.

The latency of responses to an imposed stretch may depend also by the reaction time, that is the interval between the onset of the proprioceptive signal and the time of the intended reaction. Subjects can be instructed to react to the same type of stimulus or to choice a specific response within a numbers of conditions. The first is the case of simple reaction time, which corresponds to the blocked condition in our experiments; the latter is the case of choice reaction time which corresponds to the unpredictable condition in our work. Simple reaction time for a proprioceptive stimulus may range between 60 and 155 ms [40], while the interval increases when the subject has to wait for two or more conditions. On this basis, we exclude that the process of reaction time could influence the latency or the amplitude of the short-latency reflex while it is possible that long-latency reflex and voluntary reaction may be superimposed to the reaction time [41] (see the next section).

### Effects of the load predictability on the level of muscle activation

The earliest muscle response after the movement onset was inadequate to compensate for the two loads, thus, although the finger extension slowed down, the angular excursion associated with the heavier load was larger than that observed for the lighter load. The level of muscle activation increased from short- to long-latency reflex and, in the predictable condition, long-latency EMG activity showed clear load dependence.

The different amount of activation between short- and long latency reflex is in accord with the level of EMG activity reported in most studies comparing the two muscle responses with systematic load variations [2], [8], [9], [29]. In addition, the level of long-latency response may depend on the context: our participants reacted to the perturbation with the aim to return the handle back approximately to the initial position. Several studies have shown a prevalence of long-latency reflex when the subjects were instructed to preserve a position after a perturbation instead to maintain an isometric force or not react to the perturbation [2], [29], [42]. A direct relationship between load and muscle response was also observed during voluntary movement [43], [44].

Over the interval of the long-latency reflex the significant interaction between loads and predictability conditions indicates that muscle activity is targeted to the pulling load when a quantity of anticipatory information on the load weight was provided. In fact, the gap between the two levels of EMG relating to the loads strongly decreased from predictable to unpredictable condition (Fig. 6D).

Considering the good correlation between long-latency EMG activity during unpredictable condition and the peak acceleration after the movement onset (fig. 7A–B), we conclude that the variability of this muscle response depended mainly on information associated directly with the perturbation rather than on signals provided in advance with respect to the movement onset. In this way, the control system may compensate for incorrect mechanical adaptations during the early response to the perturbation. This result is in accord with the observation reported by Pruszynski et al., [42]. These authors found a reduction of the dependence of long-latency reflex on preplanned motor strategy when the subjects were asked to maintain a reference arm position after a force applied on an unpredictable direction. Thus, the lack of changes in EMG activity with respect to the loads, observed for short- and long-latency responses in the case of the unpredictable trials, may depend on the inherent limitations of the feedback control.

The relationship between EMG response and load level improved later at the boundary between long-latency reflex and voluntary response (Fig. 6F). The partial involvement of the long-latency reflex might depend on a possible functional partition of this response as suggested by Pruszynski et al., [3]: the early portion of the response is functionally linked to the short-latency reflex while the late component is more associated with the features of the volitional response (see also [42], [45]). Against the dependence of short- and long-latency responses to the direct effect of the perturbation during unpredictable condition, the lack of correlation between long-latency EMG level and the peak acceleration during predictable condition indicates that the modulation of muscle activity was not directly associated with the perturbation when the subjects knew the upcoming load. Therefore, to explain the changes in EMG activity observed during predictable condition, we suggest that most of the signal was anticipated by using an internal representation of the perturbation dynamics encoded from previous experiences.

This scheme could be in line with the idea that the nervous system encodes physical properties of external objects and incorporates them into an internal model that predicts the sensory consequences of the mechanical perturbation (for an extensive review see [46]). This means that the nervous system tries to simulate the sensory information that will be delivered by the upcoming perturbation and uses this prediction to optimize the muscle response. Several studies have demonstrated that long-latency reflexes may be involved specifically in processes of encoding in advance the dynamic properties of a motor action [4], [16], [17], [47], [48]. For example, when a long-latency reflex is elicited during the preparatory phase of a voluntary reaching movement, it is influenced by the formation of an internal model of limb dynamics associated with the upcoming movement [4], [17], [48]. With respect to the cited studies where the long-latency reflex changed as an internal model was associated with an upcoming voluntary action, in the current experiments the potential internal model incorporated in the long-latency response would reflect the dynamics of the perturbation itself.

The amplitude of long-latency reflex and voluntary response may be affected by the reaction time [41] which is longer during unpredictable tests (two choice reaction time) than predictable condition (simple reaction time), In addition, the reaction time values showed a certain variability across subjects thus an overlap of reflex or voluntary response with the reaction time may occur early or later above 50–60 ms from the movement onset. Most of this variability depends on the level of subject attention, since other elements, such as nerve conduction velocity, synaptic transmission, neural encodings, should be stable in a well learned task. Considering that subjects were instructed to relax at the most and given the good correlation between acceleration and EMG response during long-latency reflex over the unpredictable tests, most of the variance explained by these responses should depends on the perturbation dynamics with a marginal contribution of reaction time variability.

### Effects of practice

An alternative view with respect to the formation of internal model to explain the predictive control observed in this paper could be that the adaptation of the muscle reaction to the perturbation was performed trial-by-trial in the sense that the error in the last trial modulates the change in the feedforward command for the next trial [46], [49]. Throughout this process, the error progressively reduced with an early phase marked by rapid improvements, followed by a phase in which the performance approximates a reference target much more gradually. This schema represents a common feature observed across many experimental studies on motor learning such as reaching in force fields, visuomotor adaptation and grip force adaptation (see [49]) The data reported in Fig. 5D showed no significant trend across trials, strongly suggesting that our experimental paradigm was not suitable to stimulate a learning process. Trial-by-trial learning typically requires a number of trials much higher than that used in this study and the performance may improve further when the task is recalled hours or days after. Several authors reported that an interval of hours or days may be necessary to consolidate and further improve an acquired motor ability [50]. Although it is not surprising to observe a stable level in the performance outcome of a simple stereotyped reactive action, several studies reported a certain degree of plasticity also in circuit like that serving the stretch reflex [7]. Therefore, it is possible that more number of trials and a distribution of the practice over few days could induce significant improvement in task such as that used in this work.

Based on the foregoing considerations, we believe that changes in latency and amplitude of EMG response reported in this paper would be based on internal representation, built on previous similar experiences, and rapidly transferred in the context of our task.

### How agonist-antagonist muscles activation may affect the data interpretation

The flexion of four fingers is a highly complex mechanical action that requires the coordination of a number of hand joints and muscles. We are confident that the procedures used to test and monitor EMG activity maximized the FDS contribution to the recorded signal. Nonetheless, we cannot exclude that a certain amount of EMG activity from synergist or antagonist muscles may have contaminated the signal.

One possible contamination may origin from the distribution of afferent innervation into the spinal circuits. As the fingers extended, the reflex response to muscle stretching is not necessarily limited to the muscle being stretched, but heteronymous afferent signals from Ia fibers may involve muscles of the neighboring joints [51]. Thus, in addition to muscles acting upon the same joint, wrist flexors, such as Flexor Carpi Radialis and Flexor Carpi Ulnaris, may also be activated simultaneously to the FDS. For example, wrist flexors could stabilize the wrist to provide a support to the fingers during grasp action; this can be performed by isometric contraction even if the hand is restrained by strapping. However, given the great synchronized finger movements imposed by the task, the common timing in sensory stimulation, the monosinaptic connections of Ia afferents also for heteronymous innervation, the comparable conduction velocity of motor fibers serving these muscles and the similar anatomic location, the timing of the reflex response should be the same regardless the level of contaminations. Therefore, the lag in latency observed in this paper between predictable and unpredictable conditions should not change significantly with the presence of a residual contamination from the EMG signal of muscles close to the FDS.

A later interference of other muscles than FDS, may be produced by the activation of polysynaptic circuits associated with cutaneous or type II afferences or by voluntary reactions [32], [33], [41]. These activations may affect the amplitude of EMG signal during the finger flexion and the movement completion (mainly above 50 ms). However, regardless possible contaminations by more muscles activations, the association between EMG activity and the load level found in this work remain a solid data. Thus, the idea that this result could be a consequence of a central encoding of the perturbation dynamics, should persist even if the EMG signal recorded was a combination of more muscle activations. It remains to determine the pattern of these activations: the wrist flexor should support the finger flexion, but antagonist muscles, such as the Extensor Digitorum Comunis (EDC), should restrain the overshoot flexion to optimize the end of the movement. The latter muscle synergy, known as the triphasic pattern, was reported during ballistic single-joint movement: a first burst of the agonist is followed by a burst of the antagonist and finally by a co-activation of both (see [52]). The EDC may be involved in the modulation of the overshoot flexion observed at the end of the movement. In particular, the higher level of overshoot showed during unpredictable test with respect to predictable condition (see Fig. 2 and Fig. 3C) could result from an inaccurate timing of the triphasic pattern associated with a deficit of load prediction. The organization of these muscle synergies is beyond of the interest of this paper but it can be an interesting topic for a further investigation.

Overall, we are confident that the FDS gave the main contribution to the EMG signals recorded in this paper, and that these signals may be representative of predictive-dependent commands provided by the nervous system to control a reactive grasping.

## Conclusions

The data reported in this paper indicate that when the dynamic characteristics of a rapid joint displacement are known in advance the muscle response is faster andthe muscle activity is modulated in relation to the mechanical demand. We suggest that this behavior may be associated with an internal representation of the perturbation dynamics.
